# Ventriculomegaly associated with ependymal gliosis and declines in barrier integrity in the aging human and mouse brain

**DOI:** 10.1111/acel.12184

**Published:** 2013-12-17

**Authors:** Brett A Shook, Jessica B Lennington, Rebecca L Acabchuk, Meredith Halling, Ye Sun, John Peters, Qian Wu, Amit Mahajan, Douglas W Fellows, Joanne C Conover

**Affiliations:** 1 Department of Physiology and Neurobiology, University of ConnecticutStorrs, CT 06269-3156, USA; 2 Stem Cell Institute, University of ConnecticutStorrs, CT 06269-3156, USA; 3 Department of Anatomic Pathology and Laboratory Medicine, University of Connecticut Health Center400 Farmington Avenue Farmington, CT 06030, USA; 4 Department of Diagnostic Radiology, Yale School of MedicineNew Haven, CT 06520-8042, USA; 5 Department of Diagnostic Imaging and Therapeutics, University of Connecticut Health Center400 Farmington Avenue Farmington, CT 06030, USA

**Keywords:** aging, ependymal cells, human, injury, lateral ventricle, mouse, neural stem cells, ventriculomegaly

## Abstract

Age-associated ventriculomegaly is typically attributed to neurodegeneration; however, additional factors might initiate or contribute to progressive ventricular expansion. By directly linking postmortem human MRI sequences with histological features of periventricular tissue, we show that substantial lateral ventricle surface gliosis is associated with ventriculomegaly. To examine whether loss of ependymal cell coverage resulting in ventricle surface glial scarring can lead directly to ventricle enlargement independent of any other injury or degenerative loss, we modeled in mice the glial scarring found along the lateral ventricle surface in aged humans. Neuraminidase, which cleaves glycosidic linkages of apical adherens junction proteins, was administered intracerebroventricularly to denude areas of ependymal cells. Substantial ependymal cell loss resulted in reactive gliosis rather than stem cell-mediated regenerative repair of the ventricle lining, and the gliotic regions showed morphologic and phenotypic characteristics similar to those found in aged humans. Increased levels of aquaporin-4, indicative of edema, observed in regions of periventricular gliosis in human tissue were also replicated in our mouse model. 3D modeling together with volume measurements revealed that mice with ventricle surface scarring developed expanded ventricles, independent of neurodegeneration. Through a comprehensive, comparative analysis of the lateral ventricles and associated periventricular tissue in aged humans and mouse, followed by modeling of surface gliosis in mice, we have demonstrated a direct link between lateral ventricle surface gliosis and ventricle enlargement. These studies highlight the importance of maintaining an intact ependymal cell lining throughout aging.

## Introduction

Throughout adulthood and following injury, most epithelial linings of the body display remarkable plasticity and show considerable regenerative ability due to their associated stem cell niche (Fuchs *et al*., 2004; Blanpain *et al*., 2007; Snippert *et al*., 2010). A proliferative stem cell niche, the subventricular zone (SVZ), is also maintained along the lateral wall of the lateral ventricles in the adult mouse brain (Doetsch *et al*., 1997; Tropepe *et al*., 1997; Jin *et al*., 2003; Enwere *et al*., 2004; Maslov *et al*., 2004; Conover & Shook, 2011; Shook *et al*., 2012), and SVZ stem cells can contribute to repair of the lateral ventricle epithelial lining, the ependyma, throughout aging (Luo *et al*., 2006, 2008; Conover & Shook, 2011). However, we found that while regenerative stem cell-mediated repair can address the modest ependymal cell loss found in aged mice, extensive damage to the ependyma instead results in dense surface gliosis or ‘scarring’ (Del Carmen Gomez-Roldan *et al*., 2008; Luo *et al*., 2008).

The ependyma provides a bidirectional barrier and transport system for cerebral spinal fluid (CSF) and interstitial fluid exchange (Del Bigio, 2010; Johanson *et al*., 2011; Roales-Bujan *et al*., 2012), helping to keep the brain toxicant free and in physiologic balance (Johanson *et al*., 2011; Roales-Bujan *et al*., 2012). The repercussions of periventricular gliosis instead of regenerative ependymal cell repair would be significant due to the loss of the bidirectional transport system provided by the ependymal cells lining the ventricles (Roales-Bujan *et al*., 2012). Fluid-attenuated inversion recovery MRI (FLAIR-MRI) scans of aged humans typically show ventriculomegaly with associated periventricular hyperintensities, indicative of edema (Fazekas *et al*., 1993; Sener, 2002).

Through aging insults such as infectious agents, toxic substances and trauma may result in ependymal cell loss (Del Bigio, 2010; Johanson *et al*., 2011). It is therefore important to note that the human SVZ stem cell niche, while highly active in infancy, declines dramatically by 18 months of age (Sanai *et al*., 2011; Wang *et al*., 2011; Bergmann *et al*., 2012), suggesting that stem cell-mediated regenerative replacement of lost or damaged ependymal cells is likely not an option in aged humans. As a result, loss of ependymal cell coverage and functionality would affect periventricular interstitial fluid homeostasis and transependymal bulk flow mechanisms. While the demise of the ependymal lining has been linked to hydrocephaly/ventriculomegaly associated with many neurological/psychiatric illnesses (e.g., autism, ADHD, schizophrenia) [(Palha *et al*., 2012) and references therein], the molecular and cellular mechanisms underlying ependymal cell loss and its involvement in the initiation or progression of ventriculomegaly or other disease mechanisms remain largely enigmatic. As the human brain is larger than the mouse, Cserr postulated that there would be a greater dependence on efficient clearance of interstitial solutes, particularly larger molecules in humans (Cserr, 1971), suggesting that loss of a functional ependyma would be of greater consequence to humans (Del Bigio, 2010; Johanson *et al*., 2011).

Several studies have documented the cytoarchitecture of the lateral ventricle walls in healthy young mice (Mirzadeh *et al*., 2008; Shen *et al*., 2008; Tavazoie *et al*., 2008), and our laboratory and others have extended those studies to include analysis of the ventricular lining through aging (Luo *et al*., 2006, 2008; Bouab *et al*., 2011; Conover & Shook, 2011; Shook *et al*., 2012). Here, we report that aged mice show neither periventricular gliosis nor ventriculomegaly, but instead exhibit a relatively healthy and functional SVZ stem cell niche and ependymal lining through aging. In an extensive comparison of mouse and human periventricular tissue, we reveal striking species-specific differences in the integrity and general maintenance of the lateral ventricle lining through aging. In addition, we present the first comprehensive documentation of the aged human lateral ventricle apical surface based on whole-mount preparations of portions or the entire lateral ventricle walls. Findings from human tissue analysis then informed our generation of appropriate models in mouse. We show that following extensive insult to the ependymal lining in mice, similar repair mechanisms do exist between mouse and humans, allowing further investigation into the association between ventricle surface gliosis and ventriculomegaly.

## Results

### Humans exhibit age-related lateral ventricle expansion and periventricular gliosis

**Figure 1:**
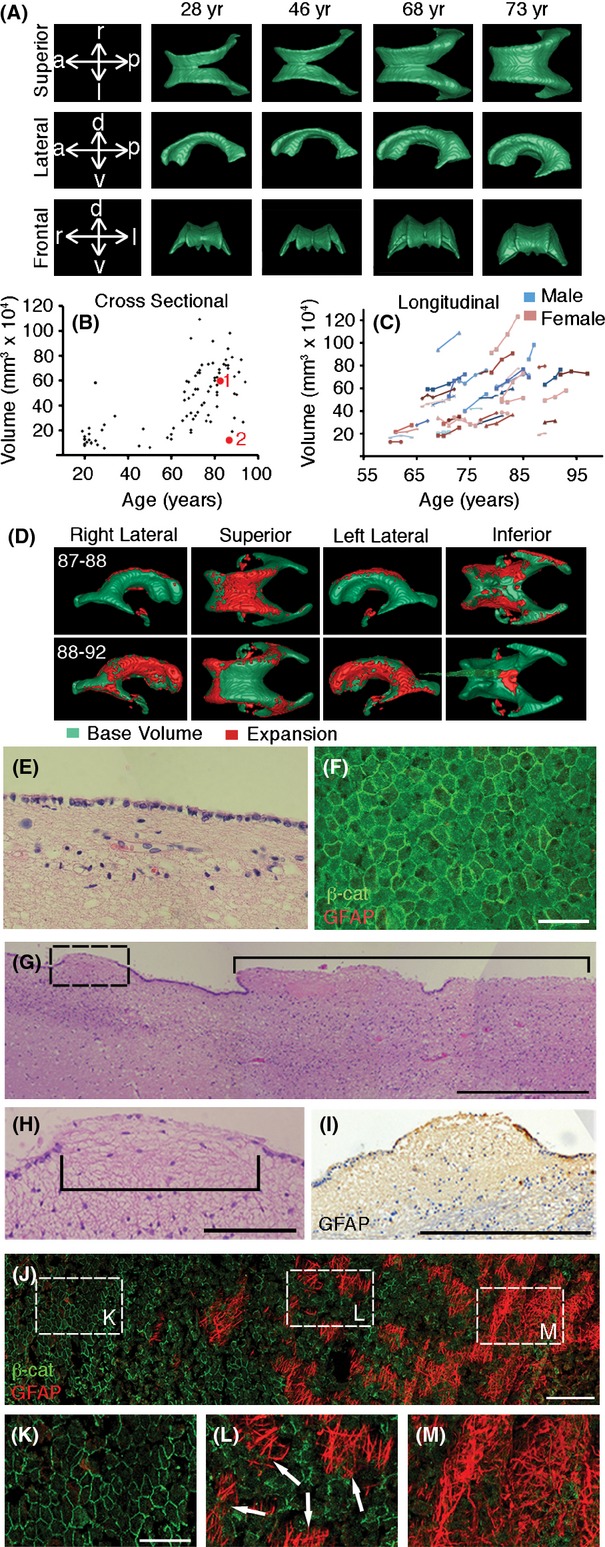
Increased ventricle volume and periventricular gliosis are typically associated with age in humans. (A) Representative MRI-based 3D reconstructions of the lateral ventricle from humans of different age groups. (B) A cross-sectional sampling of MRI scans from the Open Access Series of Imaging Studies data set (nondemented) demonstrates that increased lateral ventricle volume is associated with age. (Subject 1 and Subject 2 volumes are superimposed and denoted with red dots). (C) Longitudinal data show that expansion typically occurs over time in both males and females. (D) MRI-based lateral ventricle reconstructions show example of regional expansion (red) for two subsequent time periods. (E, F) From aged tissue (56 years old), periventricular, coronal sections stained with H&E and whole-mount preparations of the lateral wall of the lateral ventricle show regions where an intact ependymal is present. (G) H&E staining of representative coronal sections of aged tissue (77 years old) clearly show areas devoid of an intact ependymal layer (bracket). (H) Enlarged region denoted in (G) shows regions lacking ependymal cells, and (I) immunohistochemical analysis reveals that areas without an ependymal monolayer have GFAP^+^ processes at the ventricle surface and a thick hypocellular region. (J) Whole-mount preparations (tissue from 62 years old) revealed areas of intact ependyma (K) and large expanses of GFAP^+^ processes (L, M), magnification of contiguous sheets of ependymal cells (K), ependymal cells with small GFAP^+^ clusters (L), and regions of extensive gliosis (M). Scale bars, 25 μm (F); 500 μm (G, I) and 100 μm (H, K); and 200 μm (J).

Using data from the ‘Open Access Series of Imaging Studies’ (OASIS) database (Marcus *et al*., 2007) that included cross-sectional MRI data from young, middle-aged, and older nondemented adults, we examined changes to the lateral ventricles. Semiautomated segmentation using itk-snap (Snippert *et al*., 2010), followed by 3D reconstruction of the lateral ventricles using Slicer3D, revealed age-related increases in total volume (Fig. 1A,B). Based on longitudinal data from the OASIS collection, which included 150 subjects aged 60–95 years (Marcus *et al*., 2010), we observed sequential age-associated lateral ventricle volume increases in both males and females (Fig. 1C,D), similar to other previously reported studies (Scahill *et al*., 2002; Resnick *et al*., 2003). These data illustrate that ventricle expansion occurs in aging even in the absence of dementia.

To evaluate the integrity of the ependymal lining through aging, we examined aged lateral ventricle tissue samples collected by the Harvard Brain Tissue Resource Center and the University of Connecticut Health Center, Department of Anatomical Pathology (see Experimental procedures, Table S1, Supporting information). Both coronal sections and whole-mount preparations of the lateral walls of the lateral ventricles were evaluated based on 12 mm × 12 mm tissue samples. Tissue was categorized as having (i) intact ependymal cell coverage; (ii) dense areas of surface gliosis; or (iii) an intermediate phenotype consisting of a disorganized and compromised ventricle lining showing absence of cuboidal ependymal cells as indicated by diffuse or absent β-catenin staining (marker of adherens junctions) and the presence of several surface astrocytic processes (see Table S1). Typically, in subjects > 55 years old, a combination of both small and large expanses of intact ependymal cell coverage (Fig. 1E,F) and large areas devoid of ependymal cells were detected. Staining for glial fibrillary acidic protein (GFAP) indicated surface gliosis in regions where an intact ependymal lining was absent (Fig. 1G–I), and a dense array of astrocytic processes occupied an enlarged hypocellular region that typically defined the narrow subependymal zone (Quinones-Hinojosa *et al*., 2006), often creating a gliotic bulge at the ventricle surface (Fig. 1G–I).

Whole-mount preparations of the lateral ventricle provide a unique ability to examine the apical surface of the ventricle wall and have been used to observe the organization of ependymal cells and surface-projecting neural stem cells in mice (Mirzadeh *et al*., 2008). Here, we used whole-mount preparations to characterize alterations that occur to the surface ependymal cell layer (Doetsch & Alvarez-Buylla, 1996; Conover *et al*., 2000) in aged human tissue samples. Our analysis revealed both zones of contiguous ependymal cell coverage and regions of extensive gliosis (Fig. 1J–M). While these studies indicate that surface gliosis occurs in regions where ependymal cell coverage has been lost and this phenotype is prominent in tissue from aged humans, these studies provided only limited information because accompanying measurements of ventricle volumes were not available.

### Pairing postmortem MRI with histological analysis of periventricular tissue reveals association between gliosis and ventriculomegaly

**Figure 2:**
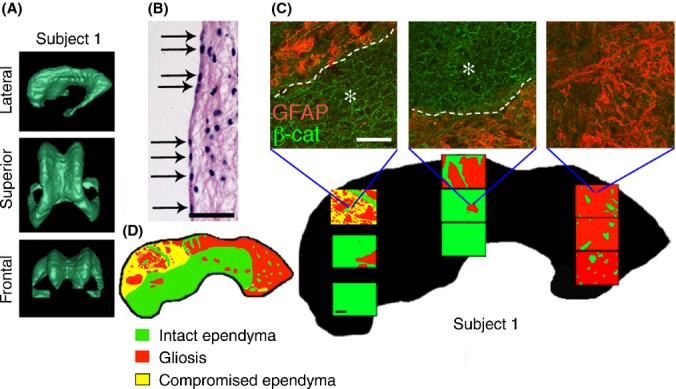
Large ventricular volume is associated with widespread gliosis at the ventricle surface in humans. (A) MRI-based 3D reconstructions of the lateral ventricle for Subject 1. (B) H&E staining of periventricular tissue revealed a compromised, attenuated ependymal cell lining of mixed cell composition, including areas devoid of ependymal cell coverage (arrows indicate separation between nuclei of ependymal cells). (C) Representative regional images from extensive immunohistochemical analysis of the ventricle surface revealed that while some areas with normal ependymal cell coverage were present (*, β-catenin indicates cell borders), large expanses of gliotic scarring at the ventricle surface (GFAP^+^ astrocyte processes) predominated. (D) Coded schematic of entire lateral wall of the lateral ventricle, with red indicating areas of astrocytic gliosis, yellow indicating a compromised ependymal lacking a distinct layer of cuboid ependymal cells, and green indicating intact ependyma. Scale bars, 100 μm (B); 40 μm, confocal image (C); 1 mm, schematic (C).

To examine directly the relationship between ventriculomegaly and glial scarring at the ventricle surface in human tissue, we paired postmortem MRI scans with histological processing of periventricular tissue. Two age-matched subjects were selected based on dramatic differences in lateral ventricle volumes. A contiguous, thin section, 3D T1-weighted MP RAGE sequence was performed on the formalin-fixed brains of the two subjects. Automatic segmentation of the lateral ventricles defined on high-resolution MRI was performed using itk-snap (Snippert *et al*., 2010) and volumes rendered using Slicer3D. 3D reconstructions showed that Subject 1 (82 years) had an age-appropriate lateral ventricle volume (59241.4 mm^3^; Subject 1 volume indicated in red on graph in Fig. 1B, see also Fig. 2A). Local microscopic features of periventricular tissue were then mapped using corresponding lateral ventricle tissue processed for histology (Fig. 2B–D). Both coronal sections (Fig. 2B) and 15 mm × 8 mm *en face* whole-mount samples (Fig. 2C) were analyzed, effectively covering the entire lateral ventricle lateral wall. Tissue samples from Subject 1 contained regions showing an attenuated ependymal cell lining (Fig. 2B), and immunohistochemical analysis of whole-mount preparations of the lateral ventricle wall revealed that while the inferior portion of the anterior and middle wall consisted mainly of intact ependymal cell coverage (Fig. 2C,D, green), ‘islands’ of dense gliosis were found scattered throughout (Fig. 2C,D, red). In the superior/anterior region, large areas of mixed composition were detected. These areas were comprised of regions of surface gliosis (red); isolated regions containing an intact ependyma (classic cobblestone appearance); and regions with attenuated cell coverage marked by the absence of clear β-catenin^+^ surface adherens junctions that define cuboid ependymal cells, some surface astrocytic processes, but no overt gliosis. An apical ventricle surface of this composition was labeled a ‘compromised’ ependymal cell lining (Fig. 2D, yellow). Along the superior (mid and posterior) and entire posterior lateral ventricle wall of Subject 1, large GFAP^+^ gliotic expanses predominated (Fig. 2C,D, red).

**Figure 3:**
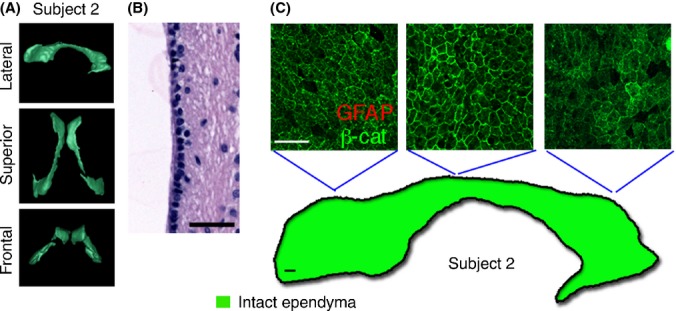
An intact ependyma is found along the entire ventricle surface in the elderly subject with a small volume ventricle. (A) MRI-based 3D reconstructions of the lateral ventricle for Subject 2. (B) H&E staining revealed a robust ependymal monolayer, and (C) immunohistochemistry of whole-mount preparations showed uninterrupted ependymal cell coverage with no surface gliosis. Scale bars, 100 μm (B); 40 μm, confocal image (C); 1 mm, schematic (C).

In contrast, MRI scans from Subject 2 (86 years) showed a ventricle volume (11279.5 mm^3^) more typical of 20- to 40-year-olds (Subject 2 volume indicated in red on graph in Fig. 1B, see also Fig. 3A). Haematoxylin and eosin (H&E) staining of coronal sections of the anterior wall revealed a monolayer of cuboidal ependymal cells, indicative of a healthy ependymal lining (Fig. 3B). Whole-mount preparations of the lateral ventricle from Subject 2 revealed intact ependymal cell coverage along the entire lateral wall of the lateral ventricle (Fig. 3C, green); we did not detect any gliosis along the lateral wall.

To examine whether the two subjects showed differences in dividing cells within the SVZ, we labeled fixed tissue samples with Ki67 to mark cycling cells. We found only a few Ki67^+^ endothelial cells; no other SVZ cells were labeled in either Subject 1 or Subject 2. As with all end-state fixed tissue samples, it is impossible to determine whether any differences in cycling cells may have existed at earlier time points for the two subjects.

The thickness and density of the astrocytic ribbon layer and hypocellular gap region of the two subjects were also compared. In regions containing an intact ependymal cell lining, no significant differences were observed (data not shown). However, in regions lacking ependymal cell coverage (Subject 1), large masses of ventricle-contacting astrocytic processes were detected increasing the thickness of astrocyte coverage from the glial ribbon to the ventricle surface.

### Age-related lateral ventricle remodeling is stem cell-mediated in mice

**Figure 4:**
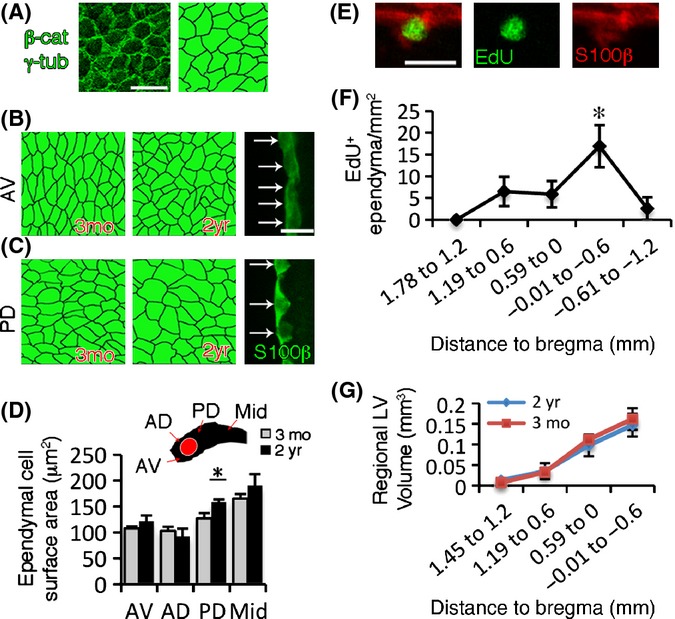
Throughout aging, mice maintain an intact ependyma, and lateral ventricle volume does not change. (A) Representative confocal image from a whole-mount preparation of lateral ventricle lateral wall and its cartoon representation. β-catenin labels adherens junctions at ependymal cell borders (green outline of cells), and γ-tubulin labels basal bodies of cilia within the apical portion of ependymal cell cytoplasm (small dots within each cell). (B) The number and size of ependymal cells lining the anterior/ventral (AV, see schematic) lateral ventricle is similar in 3-month- and 2-year-old mice. A coronal section from 2-year-old mice stained for S100β reveals a dense ependymal cell monolayer at the ventricle surface (arrows indicate ependymal cell nuclei). (C) In the region posterior/dorsal (PD) to the lateral wall adhesion (red dot on schematic), ependymal cells have a larger surface area in 2-year- vs. 3-month-old mice. Coronal section from 2-year-olds stained for S100β reveals stretched ependymal cells (arrows indicate ependymal cell nuclei). (D) Regional quantification of ependymal cell surface area (*P < 0.05, students *t*-test). (E) Following a 6-week EdU chase, newly generated ependymal-like cells (EdU^+^/S100β^+^) were observed in the ependyma of 2-year-old mice. (F) Quantification of EdU^+^ ependymal cells at different regions along ventricle surface reveals a significant increase at the PD region (−0.01 to −0.6 mm from Bregma). (G) No age-related change was detected in lateral ventricle volume for any segmented region. (AD, anterior/dorsal; AV, anterior/ventral; PD, posterior/dorsal; Mid, middle) Scale bars, 20 μm (A); 30 μm (B); 15 μm (E). Data are means; error bars are SEM.

For mice aged 3 months–2 years, whole-mount preparations of the lateral ventricle walls revealed a contiguous cobblestone organization of ependymal cells at the ventricle surface (Fig. 4A). Upon closer examination of the ependymal lining, we observed no significant difference in the apical surface area of individual ependymal cells at different regions of the lateral wall (Fig. 4B,D), with the exception of the region immediately posterior and dorsal to the location of the adhesion between the lateral and medial wall (Shook *et al*., 2012; Fig. 4C,D; red dot in schematic indicates adhesion). In mice, a narrowing (stenosis) of the lateral ventricles develops through adulthood, with the lateral and medial walls adhering to each other, generating a region often devoid of ependymal cells and an SVZ (Luo *et al*., 2006, 2008; Shook *et al*., 2012). Posterior and dorsal to the region of adhesion, we observed a significant increase in ependymal cell apical surface area (mean area for individual cells) in 2-year old compared with 3-month-old mice (Fig. 4C,D). To address whether regions of local ependymal cell stretching were associated with, and perhaps an indicator for, the generation of new ependymal-like cells (Luo *et al*., 2008), EdU birth-dating was performed on 2-year-old mice and the spatial distribution of EdU^+^ ependymal-like cells assessed (Fig. 4E,F). EdU was administered (i.p.) daily for 3 days, followed by a 6-week chase. After normalizing for regional ependymal cell apical surface area differences, we detected a statistically significant increase (*P* = 0.0320) at the ventricle surface in the number of EdU^+^/S100β^+^ cells in the region posterior/dorsal to the area of adhesion (posterior and dorsal, −0.01 to −0.6 mm from bregma, see Fig. 4E,F) containing stretched ependymal cells in 2-year-old mice (Fig. 4C,D), supporting a heightened requirement for ependymal repair in this region.

The limited 3-day introduction of EdU to mark dividing cells in the above experiments provided only a snapshot of regenerative repair to the ependymal lining in 2-year-old mice. Therefore, to investigate whether significant changes in lateral ventricle volume and surface area in mice resulted during the course of aging, we generated 3D reconstructions of the lateral ventricles from mice 3 months to 2 years of age and found that volume and surface area remained constant (3 months: volume 0.3839 ± 0.0112 mm^3^, surface area 5.5125 ± 0.2235 mm^2^; 2 years: volume 0.4106 ± 0.0950 mm^3^, surface area 5.3491 ± 0.8959 mm^2^). In analysis using defined regions, we found no significant region-specific changes in volume (Fig. 4G) or surface area (data not shown). These results indicated that throughout aging in mice, the lateral ventricles do not exhibit significant expansion. However, some local remodeling via regenerative repair does occur, in particular near sites of lateral ventricle wall adhesion and stenosis, and a relatively healthy intact ependymal lining is maintained throughout life.

### Generation of a mouse model of periventricular gliosis

**Figure 5:**
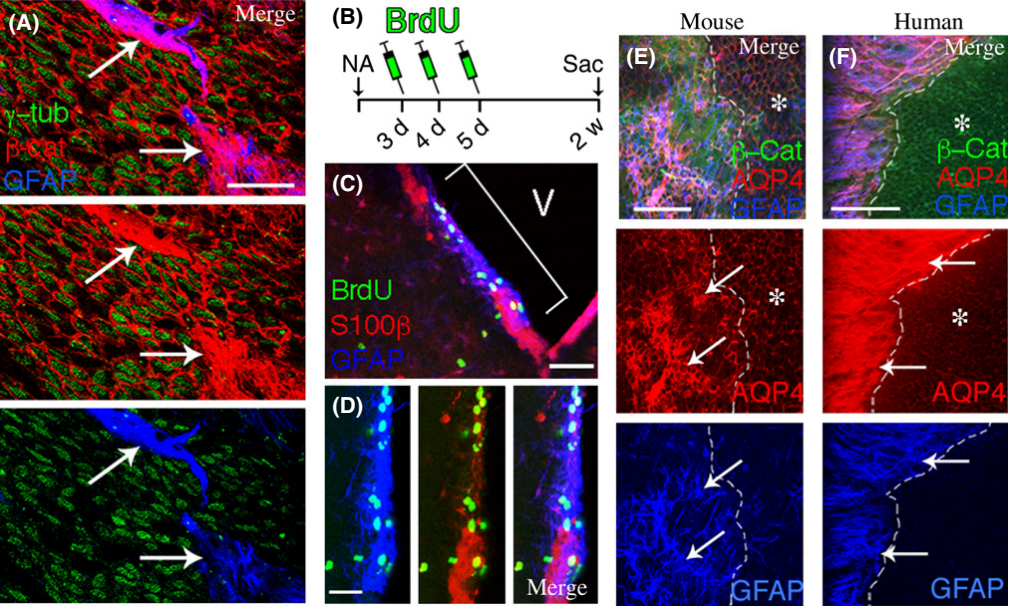
In mice, neuraminidase-induced ependymal cell denudation leads to gliosis at the ventricle surface and upregulation of AQP4, similar to AQP4 labeling in glial scars found along the ventricle wall in humans. (A) Two weeks after intraventricular injection of neuraminidase (10 ng μL^−1^), focal areas of GFAP^+^ processes were observed at the ventricle surface (arrows). (γ-tubulin marks cilia; β-catenin outlines apical membranes of ependymal cells). (B–D) Following the scheme outlined, a dense GFAP^+^ band (bracket, C), magnified in (D), containing many BrdU^+^ cells was observed in regions devoid of ependymal cells (absence of s100β). (V, ventricle) (E) Following intraventricular injection of neuraminidase (50 mU), areas of gliosis (GFAP^+^, denoted by arrows and demarcated by the dotted line) show increased expression of AQP4. Ependymal cells (*) show low levels of AQP4 staining in nonscarred regions. (F) Similarly, in human tissue, areas of surface gliosis (arrows, demarcated by the dotted line) in human tissue show increased expression of AQP4, whereas areas of ependymal cell coverage (*) are marked by β-catenin and low level expression of AQP4. AQP4 imaging was overexposed in regions of scar to show very low levels of AQP4 in intact ependymal cell layer. Scale bars, 100 μm (A, C, F); 40 μm (D); 50 μm (E).

The directionality of the relationship (cause and effect) between ventricle surface gliosis and ventricle expansion cannot be easily deduced from MRI scans and endpoint human tissue histology. To investigate this relationship, we needed to replicate in mice the phenotype of periventricular glial scarring found in aged humans. As indicated above, under normal conditions, we never observed periventricular gliosis in mice of any age, nor did we observe significant changes in ventricle volume. Whole-mount preparations of the entire lateral ventricle lateral wall consistently revealed an intact ependymal cell lining with no gliosis (see above, Fig. 4). However, damage to the ependymal cell monolayer by intraventricular injection of high concentrations of neuraminidase (100–500 ng μL^−1^) caused large stretches of ependymal denudation, resulting in extensive gliosis along the ventricle surface (Del Carmen Gomez-Roldan *et al*., 2008; Luo *et al*., 2008). Neuraminidase, found in a range of organisms including bacteria and viruses, cleaves sialoglycoproteins involved in cell recognition and adhesion (Rutishauser & Jessell, 1988; Kuchler *et al*., 1994; Figarella-Branger *et al*., 1995) and can thereby mimic ependymal cell loss due to infection [(Johanson *et al*., 2011) and references therein]. By titering the neuraminidase concentration, varying degrees of ependymal cell denudation can be generated to yield differing levels of ventricle surface gliosis (Luo *et al*., 2008). We found that 10–50 mU resulted in ventricle surface scarring similar to that typically found in aged humans (Fig. 1). Immunohistological analysis of the lateral ventricle wall showed an increase in astrocytic process density at the lateral ventricle surface in the form of scars (Fig. 5A, arrows), similar to that found along the surface of the lateral ventricles in aged humans (Figs 1J,M and 2C). No scarring or damage was detected in the third or other ventricles, indicating a direct and local effect of intraventricular injections. In addition, loss of the ependymal lining resulted in loss of the associated SVZ stem cell niche [(Luo *et al*., 2008; Jimenez *et al*., 2009; Rodriguez *et al*., 2012) and references therein]. With this mouse model, we could then investigate the connection between periventricular gliosis and ventriculomegaly and begin to dissect the cellular mechanisms that lead to ventricle surface gliosis.

To determine whether proliferating astrocytes contribute to surface gliosis, we labeled dividing cells with BrdU (three daily injections) 3 days after neuraminidase treatment (ependymal cell denudation) and then analyzed coronal sections of the lateral ventricle lateral wall (Fig. 5B–D). BrdU^+^ astrocytes were observed along the ventricle surface at sites of ependymal cell denudation, indicating reactive gliosis occurred due to extensive injury.

### Aquaporin-4 is upregulated in sites of lateral ventricle gliosis in mouse and humans

Along with ventricle enlargement, FLAIR-MRI of elderly human brains consistently reveals age-associated periventricular edema (Leys *et al*., 1990), suggesting that transependymal bulk flow is compromised. To assess periventricular scarring and compromised CSF–interstitial fluid homeostasis in our neuraminidase-induced gliosis model, we analyzed aquaporin-4 (AQP4) expression patterns. Aquaporins are water channels, and AQP4 is expressed at low levels throughout the brain on perivascular astrocytic endfeet and on the basal surface of ependymal cells (Haj-Yasein *et al*., 2011; Iliff *et al*., 2012). AQP4 expression has been found to increase during gliosis (Sofroniew, 2009). Deletion of AQP4 has been shown to reduce the effects of cytotoxic edema and improve outcome after stroke (Sofroniew, 2009; Zador *et al*., 2009). Therefore, changing expression patterns would suggest alterations to CSF–interstitial fluid regulation (Papadopoulos & Verkman, 2007; Haj-Yasein *et al*., 2011; Iliff *et al*., 2012). Following neuraminidase treatment (50 mU), we found substantially enhanced expression of AQP4 in regions of periventricular scarring in mouse tissue (Fig. 5E, left of dotted line), but not in uninjured tissue (Fig. 5E, * area of intact ependymal cells). Similarly, high levels of AQP4 were detected in human tissue at sites of glial scarring, but not where there was intact ependymal cell coverage (Fig. 5F, * intact ependyma). These studies indicate that our neuraminidase model closely replicates ventricle surface glial scarring found in aged humans.

### Extensive ventricle surface gliosis results in ventricle expansion

**Figure 6:**
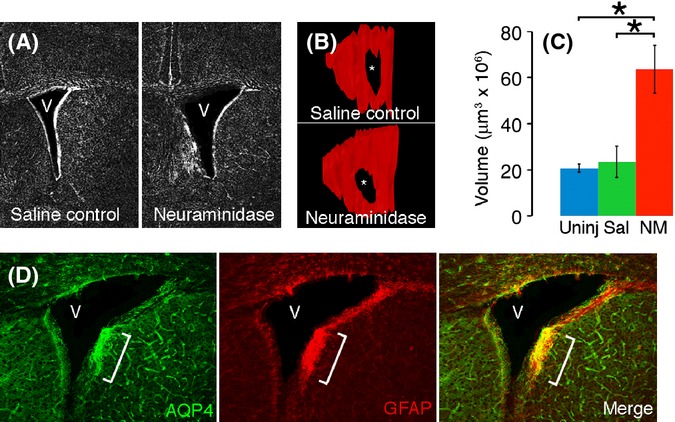
Extensive ventricle surface gliosis results in lateral ventricle enlargement in mice. (A) Two months after intraventricular injection of neuraminidase, lateral ventricles are significantly larger (V, ventricle). (B) Contours traced around the ventricles were complied to create 3D reconstructions to determine lateral ventricle volumes (* denotes adhesion between lateral and medial walls). (C) Ventricle volumes at 2 months postinjection were larger in mice that received neuraminidase compared with mice injected with saline or the uninjected littermates (*n* = 5 for neuraminidase (NA) group, *n* = 3 for saline and uninjected controls, *P < 0.03; Student’s *t*-test). (D) Immunohistochemistry confirmed gliosis and increased levels of AQP4 at the ventricle surface (bracket) and showed that it persisted 2 months after neuraminidase injection. Data are means; error bars are SEM.

We next examined the possibility that loss of ependymal cells lining the ventricle can initiate ventricle expansion, independent of neurodegeneration or other injury to the brain. Neuraminidase (50 mU, 1 μL at 500 ng μL^−1^) or saline vehicle (1 μL) was injected unilaterally into the lateral ventricle of young adult mice (3–4 months). After a 2-month period, the brains were collected and sectioned coronally. Upon visual inspection, the lateral ventricles of mice injected with neuraminidase appeared dilated compared with the lateral ventricles of mice injected with the same volume of saline vehicle (Fig. 6A). Contours were traced around the lateral ventricles and then uploaded into Neurolucida Explorer software for compilation into 3D reconstructions (Fig. 6B). Lateral ventricle volume measurements determined from the 3D models revealed that mice injected with a dose of neuraminidase sufficient to denude regions of ependymal cells similar to that found in aged humans were significantly larger than both saline-injected (*P* = 0.025) and noninjected littermates (*P* = 0.024; Fig. 6C). Lateral ventricle surface gliosis was verified using immunohistochemistry two months after introduction of neuraminidase into the lateral ventricles (Fig. 6D, bracket).

## Discussion

The ventricular system of the brain is a network of communicating cavities that encloses and circulates the CSF. Together with the blood–brain barrier, the ventricles are responsible for solute clearance from the brain. Interstitial solutes are thought to be cleared to the CSF via convective bulk flow of interstitial fluid, and a recently described ‘glymphatic’ pathway has been identified for the para-aterial influx of subarachnoid CSF into the ISF for further clearance along large-caliber draining veins that function together with and support other clearance routes (Iliff *et al*., 2012). Biochemical changes in the brain are thereby reflected in the CSF, and any alterations to the ependymal monolayer would affect efficient bidirectional transport and clearance mechanisms.

The functional units of the ventricles are the ependymal cells, which are organized as a monolayer of ciliated epithelial cells that provide both barrier and filtration functions and generate laminar flow of CSF at the ventricle surface. We examined the relationship between partial loss of the ependymal cell lining of the lateral ventricles to surface gliosis and ventricle expansion and found that significant ventricle surface gliosis can be linked directly to lateral ventricle expansion, independent of any injury or degenerative loss of brain tissue. Increased expression of the water channel AQP4 was detected in sites of glial scarring, suggesting abnormalities in CSF/interstitial fluid homeostasis and interstitial solute clearance. Our work and the work of others (Sival *et al*., 2011) suggests that the effects of replacing functional ependymal cells with a glial scar would be cumulative over time, supporting in part the progressive nature of ventriculomegaly. While we show that destruction of the ependymal lining alone is sufficient to initiate lateral ventricle expansion, it is likely that a combination of factors, such as neurodegeneration, vascular defects, and the inappropriate accumulation of soluble proteins work in concert to generate ventriculomegaly found in humans.

### Lateral ventricle ventriculomegaly and periventricular gliosis in aging humans

In aging humans, there is a strong correlation between lateral ventricle ventriculomegaly, not involving dementia or obstruction of CSF flow, and aging (Scahill *et al*., 2002; Resnick *et al*., 2003). However, the exact cause, progression, and importantly the impact to normal brain function are currently not known. Along with ventricle enlargement, FLAIR-MRI consistently reveals age-related periventricular edema, suggesting that transependymal flow is compromised. Through lateral ventricle volume measurements and 3D modeling using MRI scans from the OASIS database of nondemented humans (Marcus *et al*., 2007, 2010), we demonstrated age-associated lateral ventricle enlargement, with longitudinal data typically showing progressive increases in lateral ventricle size. Based on histological analysis of postmortem forebrain tissue samples from a population of aged subjects, we found that widespread gliosis along the lateral ventricle walls is typically found. However, these initial studies were incomplete, as access to both longitudinal MRI sequence data and tissue samples for histological analysis of the entire lateral ventricle wall was not available.

In two contrasting case studies involving brain samples from elderly subjects, we were able to analyze postmortem MRIs paired with histological tissue samples of the entire lateral ventricle. This pairing was unique as the two subjects showed significant differences in ventricle volume, which gave us the rare opportunity to examine tissue quality along the entire surface of the lateral ventricles. Our findings of regional glial scarring at the ventricle surface in the expanded ventricle sample and the complete absence of scarring and presence of an intact ependymal lining in the small ventricle sample supported our hypothesis that gliosis is associated with ventriculomegaly and not solely age. However, the sequence of events that led to gliosis at the ventricle surface and ventriculomegaly could not be deduced from this single endpoint analysis of lateral ventricle volume and surface histology. Ultimately, reconstruction of medical histories, together with a multimodal imaging paradigm including longitudinal FLAIR-MRI to show areas of edema and volume changes, and diffusion tensor imaging to show changes in major fiber tracts, would provide the level of criteria over the lifetime of the subject necessary to validate histological findings and map directionality in a correlation. So while there is no clear way to prove causality in the human cases we investigated, the information we obtained informed and guided our mouse model strategies.

### Periventricular tissue comparisons: mouse vs. humans

Due to limited availability of longitudinal, multimodal human subject data and complete histological samples of the ventricle linings, we were reliant on animal models to examine the relationship between ventricle enlargement and periventricular gliosis. Our comparison of mouse and human periventricular tissue through aging revealed some striking species-specific differences. In adult mice, the lack of significant age-associated change in lateral ventricle volume, only limited levels of ependymal cell stretching (measured as increased apical surface area), and the capacity for modest ependymal repair through SVZ stem cell-mediated regenerative replacement of ependymal cells support our finding of an intact ependymal cell lining with no surface gliosis or scarring throughout aging (Luo *et al*., 2006, 2008; Bouab *et al*., 2011). It should be noted that caged mice do not experience infection or trauma that may contribute to denudation of the ependymal lining. Periventricular gliosis occurred only in situations when we deliberately created extensive injury to the ependymal lining.

Humans, in contrast, do not appear to possess an active stem cell niche in adulthood (Sanai *et al*., 2011; Wang *et al*., 2011; Bergmann *et al*., 2012), and endothelial cells were the only proliferative cells we found within the SVZ of aged human tissue that we surveyed, regardless of ventricle size. These studies are in agreement with the work of others describing few proliferating cells in the adult human SVZ (Sanai *et al*., 2004, 2011); however, the existence and quantity of quiescent SVZ stem cells in the adult humans remains an open question (Curtis *et al*., 2007; Sanai *et al*., 2007). Interestingly, aged, nondemented humans (> 60 years), in contrast to mice, typically show age-associated ventriculomegaly and accompanying ventricle surface gliosis (Scahill *et al*., 2002; Resnick *et al*., 2003). However, some level of variability may exist between humans, as we report no periventricular gliosis along the lateral wall of the lateral ventricles in an 86-year-old who also did not show enlarged lateral ventricles. This subject died of a cardiac arrest and did not show any cortical atrophy; so, it is possible that this subject did not experience the one or several inciting events that typically result in ventricle surface gliosis in the human population. We did not find any overt difference in cycling cells or cytoarchitecture in the subependymal layer in regions containing an intact ependyma between Subject 1 and Subject 2. However, this was based on a snapshot view at the end of life. It is possible that at an earlier (maybe even mid-life) stage, there were differences in stem cell number or capacity for repair between the two subjects. The repercussions of periventricular gliosis instead of regenerative repair would seem significant, as loss of a bidirectional transependymal transport system appears to be inextricably linked to periventricular edema, loss of interstitial fluid homeostasis, and proper solute clearance (Roales-Bujan *et al*., 2012).

### Generating mouse models of periventricular scarring

Mouse studies help us to interrogate the human condition variable by variable. Here we isolated one variable, ependymal cell denudation resulting in glial scarring at the ventricle surface, to examine whether this alone could result in ventricle enlargement. Bacterial and viral infections are known to cause destruction of the ependymal lining (Johanson *et al*., 2011). Neuraminidase, a surface enzyme associated with bacteria and viruses, can result in ependymal cell denudation following severe infection. Previous studies have shown that ependymal cell denudation via injection of high concentrations of neuraminidase into the lateral ventricle resulted in glial scarring at the ventricle surface, and in some instances, SVZ cells (neuroblasts) formed clusters where there were breaks in the ependymal lining (Del Carmen Gomez-Roldan *et al*., 2008). By titering the concentration of neuraminidase injected into the lateral ventricles, we could control the degree of ependymal cell denudation (Luo *et al*., 2008) and generate levels of ventricle surface gliosis to mimic what we detected in human tissue. At the concentration and volume used in this study, we only observed glial scars at the lateral ventricle surface and never detected any damage to the ependymal lining in the third ventricle. Communication within the ventricular system was maintained. Thus, we developed an effective and clinically relevant mouse model to examine the mechanics and consequences of ventricle surface scar formation. Indeed, recent studies have identified abnormal or misexpression of adherent junction proteins as a cause for ependymal denudation in human fetuses [(Sival *et al*., 2011; Rodriguez *et al*., 2012) and references therein].

We found that the ventricle surface scars generated in mice were very similar to those observed in human tissue and showed upregulation of AQP4 expression at sites of ventricle surface gliosis. This is of particular significance as AQP4 is implicated in water uptake into brain tissue during the development of cytotoxic edema, as well as in water clearance after vasogenic edema (Papadopoulos & Verkman, 2007; Verkman, 2008; Haj-Yasein *et al*., 2011). In glial scars, the expression and localization of AQP4 constitute a neuropathological condition resulting in disturbed interstitial bulk flow and failure to clear neurotoxic solutes (e.g., Aβ, tau) [(Verkman, 2008) and references therein]. Reduced brain swelling and improved clinical outcome occurred in AQP4^−/−^ mice in a model of bacterial meningitis (Papadopoulos & Verkman, 2007), demonstrating that altered AQP4 expression may be a maladaptive response that exacerbates periventricular water accumulation. The water-transporting function of aquaporins has also been implicated in the initial formation of glial scars, affecting both the migration to the site of injury and the rate of glial scar formation [(Verkman, 2008) and references therein]. This feature may be critical and in its absence result in the inability to properly seal the ventricle lining following denudation of ependymal cells. Ultimately, AQP4^−/−^ mice in combination with our neuraminidase model should provide further mechanistic insight into the phenomenon of age-related ventricular enlargement.

## Conclusion

In mice, ependymal cell integrity and barrier function appear to be maintained, and age-related changes in lateral ventricle volume are not detected. Mice possess an active SVZ stem cell niche that is capable of limited ependymal repair throughout aging; however, the requirement for regenerative repair appears to be modest, and some stretching of ependymal cells is observed (Luo *et al*., 2006, 2008; Bouab *et al*., 2011). In contrast, aged humans typically develop ventriculomegaly and extensive glial scarring along the ventricle surface during the course of aging. Unlike other epithelial linings of the body, the regenerative replacement of the human lateral ventricle ependymal lining does not appear to be a contributing factor. As many diseases or injuries (Alzheimer’s disease, schizophrenia, traumatic brain injury, etc.) can result in ventricle enlargement, we aimed to establish some of the underlying characteristics of ventriculomegaly and found that damage to the ependymal lining resulting in lateral ventricle surface gliosis is a potential initiator of, or contributing factor for, ventriculomegaly.

Future studies incorporating noninvasive imaging for assessing ventricle lining integrity and interstitial solute movement following perturbations to the ventricle lining will be necessary to establish the time course for decline of ventricle lining health and the progression of ventricle enlargement. As we observed regional differences in the integrity of the ventricle lining in humans, it will be of particular value to determine whether regional vulnerability occurs along the ventricle surface. Together our studies highlight the importance of ventricular system health in maintaining critical barrier and filtration functions within the brain – a surprisingly understudied area in brain barrier biology.

## Experimental procedures

### Animals

Male CD-1 mice (Charles River, Wilmington, MA, USA) were aged in our vivarium; 3-month and 20- to 24-month mice were designated as young adult and aged, respectively. Animal procedures were approved by the University of Connecticut IACUC and conformed to NIH guidelines.

### Mouse brain tissue immunohistochemistry

Mice were perfused and coronal sections (50 μm) immunostained, as described (Luo *et al*., 2008). Rat anti-BrdU (1:100; Accurate Chemical, Westbury, NY, USA); goat anti-GFAP (1:250; Santa Cruz Biotechnology, Santa Cruz, CA, USA); mouse anti-GFAP (1:500; EMD Millipore, Billerica, MA, USA); rabbit anti-S100β (1:500; Dako, Glostrup, Denmark) antibodies; and Alexa Fluor-dye-conjugated secondary antibodies (Molecular Probes Life Technologies, Grand Island, NY, USA) were used. Immunostained sections were imaged on a Zeiss Axio Imager M2 microscope with ApoTome (Carl Zeiss MicroImaging, Inc., Thornwood, NY, USA), with a HAMAMATSU ORCA-R^2^ digital camera C10600 or on a Leica TCS SP2 confocal laser scan microscope (Leica Microsystems, Buffalo Grove, IL, USA). Secondary antibodies alone were used for controls.

Lateral ventricle wall whole mounts were prepared as described (Mirzadeh *et al*., 2008), and imaging protocols of whole-mount tissue preparations are as previously detailed (Shook *et al*., 2012). Whole mounts were immunostained with the following antibody combinations: mouse anti-β-catenin (1:250; BD Biosciences, San Jose, CA, USA); rabbit anti-β-catenin (1:100; Cell Signaling Technology, Danvers, MA, USA); mouse or rabbit anti-γ-tubulin (1:500; Sigma-Aldrich, St. Louis, MO, USA); mouse anti-GFAP (1:400; EMD Millipore); goat anti-GFAP (1:250; Santa Cruz Biotechnology); rabbit anti-AQP4 (1:400; Sigma-Aldrich) and imaged. The imaging focal plane was set at the ventricle surface as determined by the use of β-catenin (marks apical adherens junction proteins) and γ-tubulin (marks basal bodies of cilia) as indicators of surface depth.

### Ependymal cell apical surface area measurements: mouse

Whole mounts of the lateral ventricle lateral wall were immunostained for β-catenin and γ-tubulin and then imaged on a Leica TCS SP2 confocal microscope. The lateral ventricle surface was visualized as a continuous sheet of ependymal cells based on β-catenin^+^ (cell–cell junctions) and γ-tubulin^+^ (ciliary basal bodies) labeling (Conover & Shook, 2011). The lateral ventricle was divided into three anterior regions (anterior/ventral, anterior/dorsal, and posterior/dorsal) and middle lateral ventricle (Mid). Ependymal cells within three neighboring fields of view (150 μm × 150 μm) were counted (*n* = 3 for each age) and divided by the total area to calculate average ependymal cell apical surface area.

### EdU label retention

For examination of age-related ependymal repair, 2-year-old mice were given three daily injections (i.p.) of EdU (150 mg kg^−1^ body weight; Invitrogen Life Technologies, Grand Island, NY, USA) to label dividing cells. EdU^+^ cells in the ependymal lining were observed after a 6-week chase (time for generation of new ependymal cells; Luo *et al*., 2008). The number of EdU^+^ ependymal cells was quantified from every fourth section (12 total sections per animal, *n* = 3, +1.78 to −1.2 mm relative to Bregma) using StereoInvestigator software (MBF Bioscience, Williston, VT, USA).

### Neuraminidase and BrdU injections

Mice were anesthetized using isoflorane and positioned in a stereotaxic apparatus (Stoelting, Wood Dale, IL, USA). Neuraminidase (*Clostridium perfringens*; Roche Diagnostics, Indianapolis, IN, USA) diluted in saline (0.9% NaCl) was delivered unilaterally to the right lateral ventricle using the following coordinates relative to bregma (lateral: 0.83 mm, ventral: 2.7 mm). Neuraminidase (1 μL) was injected at a rate of 0.5 μL min^−1^. Control mice were injected with an equal volume of saline.

For cell proliferation studies, 1 μL of neuraminidase at a concentration of 10 ng μL^−1^ was injected, and 3 days later, three daily BrdU (Sigma) injections (i.p.) were given. BrdU labeling and assessment of gliosis were evaluated 2 weeks after neuraminidase injection. To study expansion resulting from substantial ependymal gliosis, 1 μL of neuraminidase at a concentration of 50 mU (500 ng μL^−1^) was injected.

### Lateral ventricle volume measurements: mouse

To determine age-related changes in lateral ventricle volume, every fourth section (10–12 coronal sections total, *n* = 3 per age, coordinates +1.78 to −1.2 mm relative to Bregma) was stained for S100β (ependymal cells) and imaged. Contour tracings of the ventricle walls and resulting 3D models were constructed using StereoInvestigator and Neurolucida Explorer (MBF Bioscience). To evaluate expansion, all sections from most anterior to the point when the third ventricle became visible were used.

### Human brain tissue histology/immunohistochemistry

Postmortem human brain tissue (hemispheres and slices) was obtained from the Harvard Brain Tissue Resource Center (McLean Hospital) and UCHC Department of Anatomic Pathology and Laboratory Medicine. Tissue was fixed in 10% formalin (minimum 2 weeks, maximum 6 weeks) and rinsed thoroughly (0.1 m PBS), and whole mounts of the lateral ventricle lateral wall were processed for immunohistochemistry, as described above. Whole mounts of all regions of the lateral ventricle (Subject 1: 36 15 mm × 8 mm sections and Subject 2: 18 15 mm × 8 mm sections) were prepared. Coronal sections were taken from the superior-most region of the anterior lateral ventricle to investigate the integrity of the ependymal lining and SVZ.

For immunoperoxidase labeling, 8-μm paraffin sections were processed using a Bond Max autostainer (Leica Microsytems). Haematoxylin and eosin staining was performed as described (Luo *et al*., 2008).

### Lateral ventricle volume measurements: humans

For evaluation of lateral ventricle volumes across age, high-resolution MRI sets were obtained from the OASIS database (www.oasis-brains.org; Marcus *et al*., 2007, 2010). Only male and female subjects remaining nondemented (clinical dementia rating < 0.5) throughout the length of the study were analyzed (Marcus *et al*., 2007). MRIs of younger individuals (nondemented subjects aged 20–34 years) were obtained from Yale Medical School. In total, 70 individuals were analyzed in six different age groups: 19–39 (*n* = 23), 40–69 (*n* = 6), 60–69 (*n* = 10), 70–79 (*n* = 13), 80–89 (*n* = 13), and > 90 (*n* = 5). The lateral ventricles were defined on high-resolution MRI T1-weighted images using a semiautomated 3D segmentation tool, itk-snap (www.itksnap.org; Snippert *et al*., 2010). Preprocessing of the images was performed prior to automatic isolation of the lateral ventricle, including setting an upper threshold for image intensity at 30%. For automatic segmentation, lateral ventricle expanding balloon force was set to 3.0, detailed curvature force was set to 0.20, and 125 iterations at step size 1 was sufficient to isolate the lateral ventricle. Volumes were rendered using Slicer3D (www.slicer.org). Longitudinal MRI sets (OASIS) were used from healthy subjects that underwent MRI scanning in three or more sessions spanning multiple years (Marcus *et al*., 2010).

### PostMortem MRI: human

Postmortem MRI was performed using a 1.5-tesla MR unit (Siemens Avanto; Siemens Medical Solutions, Malvern, PA, USA). A contiguous thin section (1.3 mm), 3D T1-weighted MP RAGE sequence was performed on the formalin-fixed brains of two individual subjects. Subject 1 (female, 82 years) was diagnosed with Alzheimer’s disease and died of pneumonia, and Subject 2 (male, 86 years) died of cardiac arrest. Lateral ventricle volumes were processed and rendered, as described above.

### Statistical analysis

Results are reported as mean ± SEM. Statistical analysis (GraphPad Prism software, www.graphpad.com, or Excel, www.microsoft.com) across multiple ages was assessed using one-way anova with Bonferroni’s multiple comparisons post-test; between ages, using a two-tailed unpaired Student’s *t-*test; and between neuraminidase injected and controls, using a two-tailed Student’s *t-*test. Significance was set at *P* < 0.05.

## Supporting Information

**Table S1.** Summary of tissue analysis: ependymal coverage vs. scarring.
