# Supplementary figures and images for: Ventriculomegaly associated with ependymal gliosis and declines in barrier integrity in the aging human and mouse brain
Source: Aging Cell. 2013 Dec 17;13(2):340–50. doi: 10.1111/acel.12184 (PMC3954884; doi:10.1111/acel.12184)

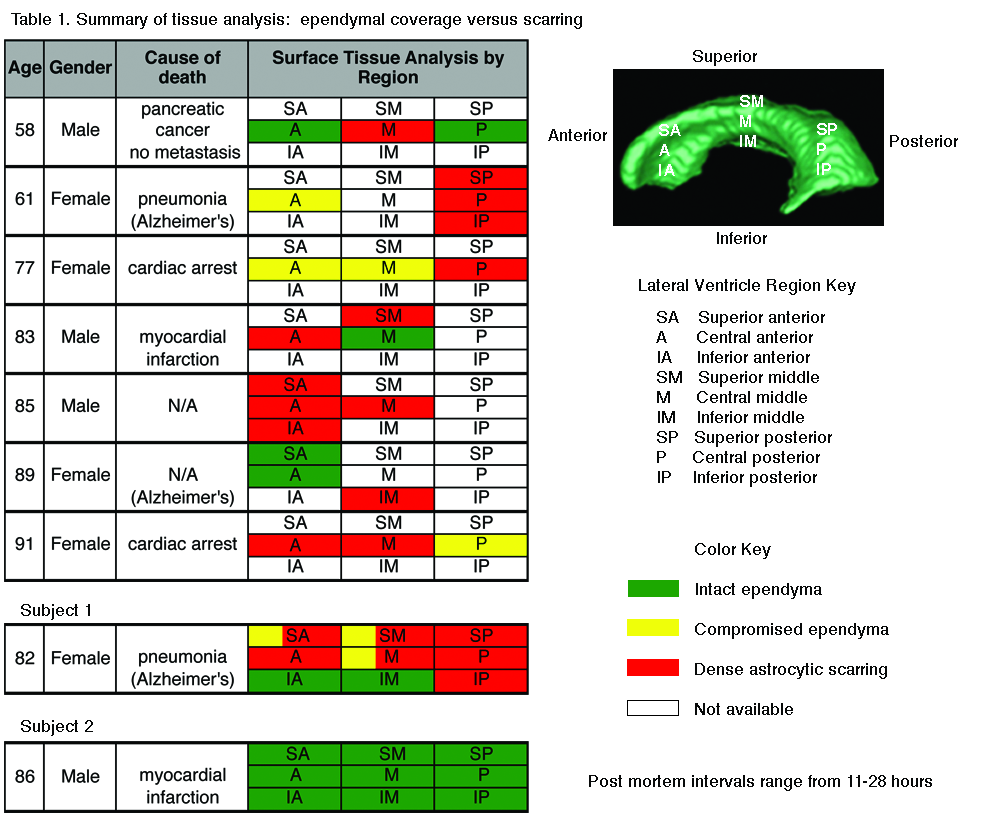

Supplement: Supplementary file 1 — Table S1. Summary of tissue analysis: ependymal coverage vs. scarring. [file acel0013-0340-sd1.tif]
